# Feasibility Study of Using Alternating Current Excitation to Obtain Electrodermal Activity with a Wearable System

**DOI:** 10.3390/s25123603

**Published:** 2025-06-08

**Authors:** Juan David Romero-Ante, Juan Sebastián Montenegro-Bravo, José María Vicente-Samper, Vicente Manuel Esteve-Sala, Miguel Ángel de la Casa-Lillo, José María Sabater-Navarro

**Affiliations:** 1 Institute of Bioengineering, Miguel Hernández University of Elche, 03202 Elche, Spain; j.romero@umh.es (J.D.R.-A.); juan.montenegro@goumh.umh.es (J.S.M.-B.); j.sabater@umh.es (J.M.S.-N.); 2 Department of Civil Engineering, University of Alicante, 03690 San Vicente del Raspeig, Spain; jm.vicentesamper@ua.es; 3 Department of Software and Computer Systems, University of Alicante, 03690 San Vicente del Raspeig, Spain; vesteve@ua.es

**Keywords:** electrodermal activity (EDA), skin conductance level (SCL), direct current (DC), alternating current (AC), conductance, susceptance, biomechanical systems, wearable systems

## Abstract

This study investigates the feasibility of using a wearable system with full-wave alternating current (AC) excitation to measure electrodermal activity (EDA). Typically measured using direct current (DC) excitation, EDA is often affected by signal drift due to electrode–skin polarisation. To address this, a portable device was developed that applies fixed-amplitude, full-wave AC signals and records EDA under controlled conditions. The electrical behaviour of the skin was also simulated using a multilayer model to analyse current propagation at different frequencies. The experimental procedure was conducted with ten healthy participants under controlled conditions. Two stages were carried out: the first compared the similarity of the skin conductance level (SCL) between DC and half-wave alternating current (AC) signals; the second analysed signal stability and skin response at full-wave AC excitation. Compared to DC, full-wave AC excitation demonstrated reduced signal drift, greater temporal stability, and enhanced measurement of the skin’s capacitive response. These findings support the adoption of AC excitation for EDA measurement, especially in ambulatory and real-time biomechanical applications where signal reliability and stability are essential.

## 1. Introduction

Electrodermal activity (EDA) is based on the measurement of skin impedance or electrical conductance. It is a commonly used parameter for assessing physiological and emotional states, such as stress levels, and provides relevant information in situations of tension or anxiety [1,2]. Furthermore, EDA is employed in user experience studies to analyse individuals’ emotional responses when interacting with different products, interfaces or environments [3].

Changes in skin conductance, measured in microsiemens (µS), are determined by measuring the electrical resistance between two electrodes placed on the skin. The resulting signal consists of two main components: the skin conductance level (SCL) and the skin conductance response (SCR). SCL reflects basal sweat glands activity in the absence of external stimuli and is calculated as the average of measurements taken at rest [4]. In contrast, SCR evaluates transient changes in conductance induced by specific stimuli, such as emotional or cognitive events [5]. These changes appear in the phasic component of the EDA signal, manifesting as peaks superimposed on the SCL that return to baseline over time [4].

SCL has been employed in the assessment of user adaptation and experience in biomechanical and rehabilitation systems [6,7], where the analysis of the tonic component of the EDA signal has enabled the quantification of physiological response changes in subjects. Based on this signal, control strategies have also been developed to enable the dynamic adaptation of robot-assisted therapies, particularly in virtual reality environments [8]. SCR has been widely used in emotion recognition systems linked to specific events, as it reflects physiological activation patterns in response to situations involving stress, fear, or calmness, thus providing valuable insights into individual behaviour [9,10,11]. Moreover, both SCL and SCR have proven to be useful tools for monitoring neurological disorders, such as peripheral neuropathy (PN), by facilitating the tracking of disease progression [12,13,14].

However, the effectiveness of these applications depends not only on how EDA is interpreted, but also on the reliability of the signal acquisition process. The exosomatic measurement of EDA is usually performed by voltage or direct current (DC) excitation due to its instrumental and analytical simplicity [15]. This method has a limitation related to the polarisation effect on the electrode–skin interface [5]. The accumulation of charges on the electrodes generates an electromotive force (EFM) that induces a voltage opposite to the applied signal, affecting the reference potential and introducing variability in the measured conductance values. Although this effect is not always immediate, it becomes increasingly significant over time, especially during prolonged recordings, where it can lead to substantial signal distortion [5]. This results in signal drift, which does not reflect actual changes in tissue or glandular activity but rather artefacts caused by electrode polarisation. This drift affects the stability of the measurement and makes it difficult to obtain reliable conductance values. In this case, the use of alternating current (AC) mitigates this problem, as the cyclic inversion of polarity minimises the induced EMF and reduces electrode polarisation [5].

In addition, the skin exhibits capacitive behaviour, allowing it to store and release electrical charge. This effect depends on the structure of the epidermis and dermis, where sweat glands modulate EDA [16]. When an AC signal is applied, cell membranes behave as capacitors, allowing the current flow to reverse in each cycle [5]. This process results in continuous charging and discharging, enabling the measurement of both conductance and susceptance, the latter being associated with the capacitive component of the skin. In contrast to DC excitation, which primarily captures the electrical potential related to the stratum corneum [17], AC-based measurements allow for a more comprehensive analysis of the skin’s bioelectrical state, providing a more accurate reflection of its physiological response.

Studies such as [18] have explored the use of unipolar AC signals for EDA measurement and have demonstrated their effectiveness in assessing sweat levels across different body areas. These measurements are obtained using a single active electrode and a common reference electrode placed elsewhere on the body, which enables the evaluation of sweating patterns. Similarly, ref. [17] compared EDA measurements using AC and DC excitation signals, finding a high correlation between both methods when using a frequency of 20 Hz in AC. Although the AC signal used was half-wave, i.e., without a negative component, the results support the validity of this methodology and position it as a promising alternative to the traditional DC technique. However, further studies are required to confirm its superiority. In this context, ref. [5] suggests that further research in this area will allow a deeper analysis of the resistive and capacitive components of the skin, providing key information about its bioelectrical behaviour. Furthermore, (p. 84, [19]), highlights that the use of a broad spectrum of AC frequencies could enhance the understanding of the electrical properties of the electrodermal system, facilitating the correlation between the tonic and phasic components of the EDA signal.

Despite these advances, previous studies have not fully overcome the key limitations of DC excitation, such as signal drift and electrode–skin polarisation noise. While half-wave and unipolar AC signals show promise, they lack the polarity inversion necessary to mitigate these effects, resulting in unstable measurements. Although some commercial and research systems already use AC-based EDA acquisition, these systems typically rely on current-controlled signals with dynamic adjustment [20]. In contrast, the present study proposes and validates the use of fixed-amplitude, full-wave AC excitation to focus on its effect on signal stability and skin interaction under controlled conditions.

This article presents a study on the electrical behaviour of the skin under full-wave AC excitation, which includes both positive and negative cycles. A custom-developed wearable system was designed to apply this excitation and acquire EDA signals. The primary objective of the study is to compare AC and DC excitation methods, with particular emphasis on the ability of full-wave AC to reduce signal drift caused by electrode–skin polarisation and to improve baseline stability. Additionally, the study explores the capacitive characteristics of the skin through electrical modelling and frequency-domain analysis. The methodology combines finite element simulations, hardware development, and experimental validation with human participants. The results section presents the findings obtained from the simulations and experiments. Finally, the advantages of using AC excitation compared to traditional DC EDA measurement methods are discussed.

## 2. Materials and Methods

### 2.1. Electrical Properties of Skin

Human skin, due to its multilayered structure and the presence of active sweat glands, can be modelled electrically as a system with resistive and capacitive properties, represented by combinations of resistors and capacitors (p. 64, [19]). The stratum corneum, which has no living membranes, acts as a sponge, absorbing and releasing water in response to environmental moisture. Increased perspiration increases its hydration, which modifies the skin’s resistance (p. 65, [19]).

Traditional electrical models, such as the one proposed in [21], describe the resistive interaction between the skin and sweat glands. In these models, a fixed resistor represents the stratum corneum, while a variable resistor models the sweat gland ducts. However, these purely resistive models are only applicable to EDA measurements using DC signals. To address this limitation, an improved model was proposed in [22], incorporating a capacitor in parallel with the epidermal resistance, allowing for the characterisation of impedance (*Z*) and the influence of sweat glands on the stratum corneum. With this addition, the application of AC signals induces a continuous charge and discharge of the capacitor, helping to mitigate the polarisation effect on the contact of the electrode–skin interface [17].

#### AC Excitation Signal for EDA Analysis

Alternating voltages, characterised by their periodic variation in magnitude and direction, such as sinusoidal signals, are defined by their voltage amplitude (*A*) and frequency (*f*). Mathematically, they can be expressed as shown in Equation (1):

(1) $$V_{AC}\left(t\right)=Asin\left(2\pi ft\right)$$

When current and voltage are measured at the terminals of a capacitor, a phase shift between both signals is observed. The current reaches its maximum value when the voltage is zero and becomes null when the voltage is at its peak. This phase shift is described by the phase angle (ϕ), which allows for the analysis of the capacitor’s ability to store and release energy. In the context of EDA response analysis, phase variation provides key information about the capacitive properties of the skin (p. 56, [19]).

Admittance (*Y*), defined as the inverse of impedance (*Z*), describes the ease with which electric current flows through a system under an AC excitation signal. Based on the calculation of ϕ, admittance can be decomposed into its two main components: conductance (*G*) and susceptance (*B*), corresponding to the real and imaginary parts, respectively. Conductance reflects the ability of the system to conduct current effectively, analogous to the behaviour observed in circuits excited with DC signals. In contrast, susceptance characterises the response of reactive elements, such as capacitors, capturing the capacitive properties of the skin. In this work, the definitions presented in (p. 59, [19]) are used to characterise both the resistance and the energy storage capacity of the skin under AC excitation, as established in Equations (2) and (3), respectively. This approach enables a more comprehensive assessment of its bioelectrical properties.

(2) G(f)=Y(f)cos(ϕ(f))

(3) B(f)=Y(f)sin(ϕ(f))

### 2.2. Simulation of the Skin’s Electrical Response to AC Excitation Signals

To analyse the current distribution across the different layers of the skin and evaluate the interaction of the AC excitation signal, a simulation based on a multilayer electrical model of the skin tissue has been developed. This model represents the skin as a system with different electrical properties in each of its layers: stratum corneum, epidermis, dermis and subcutaneous tissue, considering their respective characteristics of electrical conductivity (σ) and relative permittivity (ϵ).

The study was carried out using COMSOL Multiphysics 6.0 [23], a simulation software based on the finite element method (FEM). In this case, the electric current module was used, which allows the calculation of current density based on the electrical conductivity and relative permittivity of each skin layer. The analysis was performed in the frequency domain, enabling the evaluation of the tissue’s response to AC signals at different frequencies.

A two-electrode stimulation configuration was used for the simulation: one with a positive excitation terminal and the other connected to ground. This scheme allowed the degree of interaction of the current between the two electrodes and its propagation through the different skin layers.

The current density is calculated considering both the real and imaginary components. The real component is related to the electrical conductivity of each skin layer and the applied electric field (*E*), expressed in Equation (4):

(4) $$J_{\mathrm{real}}=\sigma \cdot E$$

On the other hand, the imaginary component of the current density is related to the frequency (*f*) of the AC excitation signal and the electrical displacement (*D*). The latter is defined as the product of the vacuum permittivity ($\epsilon_{0}$), the relative permittivity (ϵ) and the electric field, according to Equation (5):

(5) $$D=\epsilon_{0}\cdot \epsilon \cdot E$$

The imaginary part of the current density is defined by Equation (6):

(6) $$J_{im}=j\cdot w\cdot D$$

Finally, the total current density is computed by combining the real and imaginary components to form a complex quantity. The modulus is then used to obtain its overall magnitude. This represents the total current density flowing through the tissue under AC excitation.

The geometric model of the skin was built according to the thickness definitions given in [24,25]. For the electrical characterisation of the tissue, the values of electrical conductivity and relative permittivity reported in [26] were used, where the specific parameters of each skin layer are presented as a function of frequency (1 Hz, 100 Hz and 1 kHz). These values are based on the dielectric property characterisation results described in [27]. Regarding the electrode configuration, the default material for silver (Ag-Silver) defined in COMSOL Multiphysics was chosen.

In the simulation, an alternating signal of ±1.65 V (total amplitude of 3.3 V) was used. It can be generated by a microcontroller using pulse width modulation (PWM) or a digital-to-analogue conversion (DAC) output. This choice enables the analysis of the penetration depth and interaction of a signal that can be implemented in real electronic systems, providing a practical reference for its effect and interaction with the different layers of the skin.

**Figure 1:**
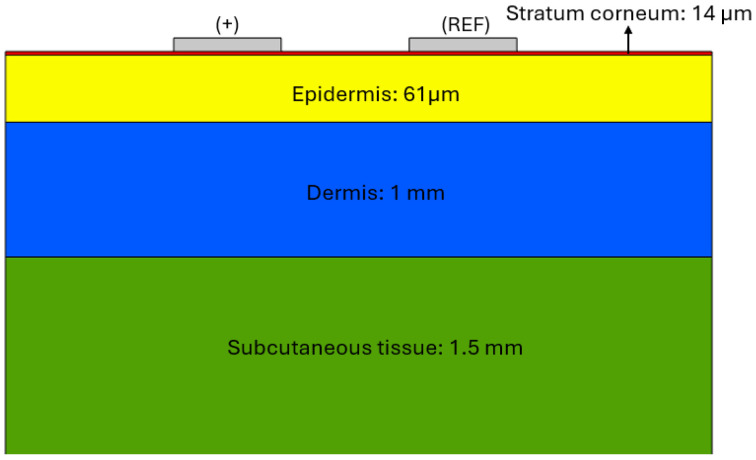
Two-dimensional model of the skin used for simulation in COMSOL. The electrodes are shown at the top: the positively polarised electrode (+) and the reference electrode (REF). The skin layers are represented by colours: stratum corneum (red), epidermis (yellow), dermis (blue), and subcutaneous tissue (green).

Figure 1 shows the structural model of the skin used in the COMSOL simulation, where its different layers and sizes are clearly distinguished: stratum corneum (red), epidermis (yellow), dermis (blue) and subcutaneous tissue (green). The upper part shows the model of the stimulation electrodes: the sign (+) indicates the positively polarised electrode, and the other one corresponds to the reference electrode (REF), both used for the application of the excitation signal.

The dermis contains the highest concentration of sweat glands, particularly on the palms, soles and forehead [28]. In the context of EDA, eccrine glands, which play a key role in thermoregulation, are of particular interest, as their activity varies in response to emotional and cognitive stimuli [29]. Since these glands affect the skin’s electrical conductivity, it is essential to analyse the penetration and propagation of current within the dermis under an AC excitation signal. Their capacitive behaviour influences the dielectric response of the tissue, making it crucial to evaluate the interaction of the AC signal with the dermis through simulation. This analysis determines the degree of penetration and its impact on EDA measurement.

**Figure 2:**
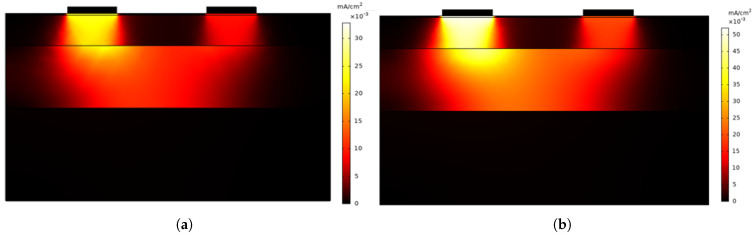
(**a**) Current density distribution in the skin for an AC excitation signal of 1 Hz frequency. (**b**) Current density distribution in the skin for an AC excitation signal of 100 Hz frequency.

Figure 2a,b show the current density distribution through the different layers of the skin when an AC excitation signal with a frequency of 1 Hz and 100 Hz is applied, respectively. The primary difference between both cases lies in the intensity of the current density and its propagation.

In both simulations, the current distribution follows a similar pattern, with the maximum intensity concentrated in the region below the electrodes. However, at 100 Hz the current density is higher, reaching peak values close to 50 mA/cm², whereas at 1 Hz the peak values are lower, around 30 mA/cm². This confirms that at higher frequencies, the impedance—mainly determined by the permittivity of each skin layer—decreases, allowing a greater current flow.

The simulation results show that when an AC excitation signal is applied at 1 Hz and above, the current reaches the dermis where the sweat glands are located. This suggests that in this frequency range it is possible to obtain relevant information about the capacitive component of the skin and thus to evaluate the associated susceptance variation. In addition, the penetration of the current into the different layers of the skin, together with the constant exchange of polarity of the excitation signal, helps minimise the effects at the electrode–skin interface, improving the measurement stability.

### 2.3. Portable Device for EDA Measurement with AC Excitation

In this work, the design and development of a portable device for measuring EDA using a full-wave AC excitation signal, i.e., with both positive and negative components, is presented. The device consists of an excitation signal generation and control stage, as well as a data acquisition and transmission stage. The components and operation of the system are described below.

#### 2.3.1. Device Design

Traditional measurement systems using direct current (DC) excitation signals [14,30] are based on the determination of the voltage drop across the skin resistance measured by two electrodes. In addition, these systems incorporate operational amplifiers to amplify and modulate the output signal to improve its interpretation.

On the other hand, the device proposed in this work is characterised by the use of a full-wave AC excitation signal. The inclusion of this type of signal introduces significant changes in the instrumental design compared to conventional systems.

**Figure 3:**
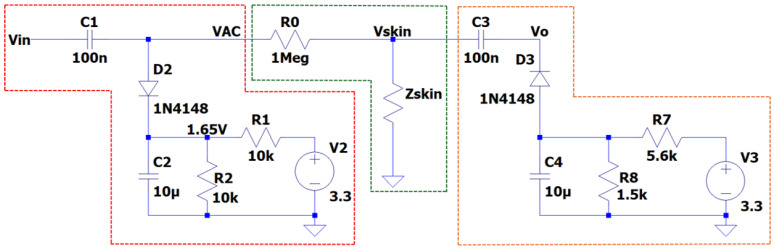
EDA measurement circuit using AC excitation. In red, the stage for the generation and conditioning of the excitation signal is shown; in green, the measurement of skin resistance; and in orange, the signal conditioning for subsequent digitisation.

Figure 3 shows the circuit design for EDA measurement using AC excitation. It highlights three main stages: in red, the generation and conditioning of the excitation signal; in green, the measurement of skin resistance; and in orange, the signal conditioning for subsequent digitisation.

$V_{in}$ is a pulse width modulation (PWM) signal generated by the microcontroller that oscillates between 0 and 3.3 V. In the first stage of the circuit (highlighted in red), a level-shifting and DC biasing stage is used to offset the AC signal symmetrically around a 1.65 V reference voltage. The diode D2 clamps the signal to protect it from exceeding the supply voltage, while the voltage divider sets the DC reference voltage for biasing the signal. Consequently, the excitation signal at node VAC transforms into a full-wave AC waveform centred around 1.65 V. When coupled through the capacitor, this waveform oscillates around 0 V with an amplitude of ±1.65 V.

The second stage of the circuit corresponds to the measurement of the skin impedance ($Z_{skin}$) through two electrodes in contact with the skin, using an AC excitation signal ($V_{AC}$). The resistor $R_{0}$ acts as a current limiter, ensuring that the excitation current applied to the skin does not exceed 20 µA, thus complying with the safety standards established for medical devices in contact with the human body [31]. The developed system is capable of recording conductance variations within a range of 0.2 to 100 µS, which covers the typical interval according to the specifications of the commercial Shimmer3 GSR+ device [32]. This sensitivity range enables accurate detection of both the tonic and phasic components of the EDA signal.

The voltage drop recorded across the skin, $V_{skin}$, depends on the relationship between $R_{0}$ and $Z_{skin}$. Thus, the system can be modelled from the expression in Equation (7):

(7) $$V_{skin}=V_{AC}\left(t\right)\cdot \frac{Z_{skin}}{R_{0}+Z_{skin}}$$

where the skin impedance is defined according to Equation (8):

(8) $$Z_{skin}=\frac{V_{skin}\cdot R_{0}}{V_{AC}\left(t\right)-V_{skin}}$$

The final stage of the system adjusts the $V_{skin}$ signal to an optimal range for acquisition by the analogue-to-digital converter (ADC) of a microcontroller. For this purpose, a positive clamper diode circuit is implemented, which shifts the signal into the positive range, allowing the $V_{o}$ output to be correctly captured and processed.

#### 2.3.2. System Control, Communication and Technical Features

The measurement equipment includes a Teensy 3.2 development board [33] equipped with a 32-bit ARM Cortex-M4 microcontroller, manufactured by STMicroelectronics, which runs at 72 MHz, with 64 KB of RAM, 256 KB of flash memory and an ADC with up to 16-bit resolution, ensuring efficient handling of variables and registers. This board manages the operation of the system, including the generation of the supply voltage Vi, an alternating signal between 0 and 3.3 V, with a frequency that can be adjusted according to the measurement requirements. To achieve this, a PWM signal with variable frequencies is used. Additionally, the Teensy generates the reference voltages used in the clamper circuits, allowing proper adaptation of the signals for measurement acquisition and processing.

On the other hand, the board receives the signal Vo through an analogue input, corresponding to the system’s output voltage, from which the skin impedance is calculated. The data is transmitted via UART serial communication to a central computer, which is responsible for storing and processing the information. The connection between the Teensy and the computer is established via USB, which facilitates data transmission and provides the necessary 5 V power for its operation.

The system operates at a sampling rate of 20 Hz, which is sufficient to accurately capture the characteristic variations of the EDA signal. A Python 3.12.10-based user interface is used to visualise the signal in real time, manage data acquisition and facilitate user interaction. The acquired data is stored in text files on the host computer for traceability purposes. These raw data files are then post-processed in MATLAB R2024a [34], where signal processing and statistical analysis are carried out.

In addition to signal generation and communication, the system was developed with portability and safety in mind. The complete hardware prototype, which includes the signal conditioning and excitation circuits, has a compact footprint of around 38 mm × 50 mm, enabling integration into wearable setups. It is powered via a 5 V USB connection and has a typical current consumption of around 40 mA, resulting in an estimated power usage of 200 mW. The device is compact, has low power consumption and is suitable for physiological monitoring. For user convenience, the device includes a 3.5 mm audio jack input for connecting the electrode cables, enabling them to be easily and securely attached to the skin surface.

**Figure 4:**
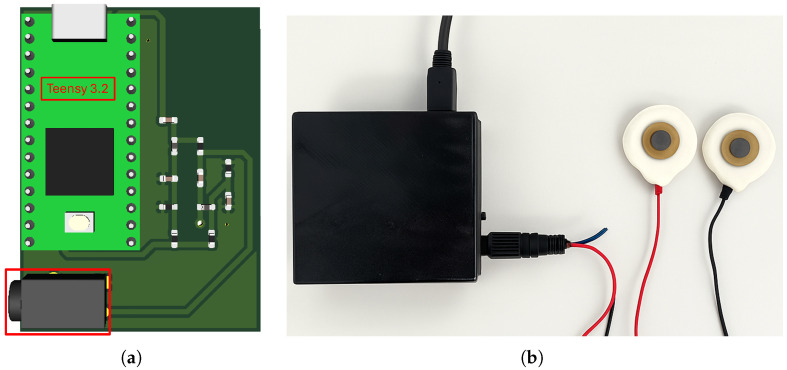
(**a**) PCB layout of the EDA acquisition system developed based on a Teensy 3.2 board. The red box highlights the 3.5 mm jack connector that is used for the electrode interface. (**b**) Final prototype, complete with a protective enclosure, a USB power and data connection, and electrode cables that are attached via the jack connector.

Figure 4a presents the PCB layout, highlighting the Teensy 3.2 board and the jack-type connector used for the electrode interface, Figure 4b shows the prototype in a compact housing with USB power and data connections, as well as electrode cables for signal acquisition.

#### 2.3.3. Excitation Signal in AC

The system generates a full-wave AC excitation signal from a positive-going PWM signal. This method was chosen by utilizing one of the pins of the development board, as it provides a cleaner signal with a lower noise level compared to one generated through a digital-to-analogue converter (DAC). The initial PWM signal has a maximum amplitude of 3.3 V and a 50% duty cycle. It is then subjected to an adaptation and shifting process through the clamper diode circuit described in Section 2.3.1. This ensures that the voltage supplied to the skin through the electrodes is full-wave alternating.

The excitation frequency in AC signals should be selected according to the objectives of the analysis. According to [35], high frequencies (>1 kHz) are suitable for detecting rapid changes, whereas lower frequencies are more effective for assessing the sensitivity of conductance related to sweat gland activity. In [17], frequencies below 100 Hz are recommended, highlighting the 10–20 Hz range as ideal. Frequencies between 10 and 100 Hz allow for a more precise analysis of sudomotor activity; however, frequencies near the power line frequency should be avoided to simplify signal filtering.

In this context, following the mentioned recommendation, this study employs positive-wave AC excitation signals at a frequency of 20 Hz to analyse the degree of correspondence between this measurement and the traditional DC excitation method. Likewise, for full-wave AC excitation, frequencies of 5 Hz, 20 Hz, 70 Hz, and 100 Hz were used, excluding 50 Hz because it coincides with the power frequency, which could introduce interference and make the signal analysis difficult. This selection of frequencies enables the evaluation of signal stability and the characterisation of the skin’s electrical properties as a function of the applied excitation frequency.

### 2.4. Signal Processing

In this section, the processing applied to the EDA signals obtained from the system is detailed. First, the filtering process is described, which aims at eliminating possible interferences and improving the signal quality. Then, the decomposition of the signal is discussed, differentiating its conductance and susceptance components.

#### 2.4.1. Signal Filtering

The EDA signal filtering is performed using a moving average filter, which calculates the average of values within a sliding data window along the signal. This helps attenuate high-frequency fluctuations and reduce noise, preserving the overall trend and ensuring temporal smoothing without losing relevant information.

For this process, a window of 20 samples is used, equivalent to one second of data, as it matches the system’s sampling frequency. This configuration ensures adequate noise elimination while preserving the significant changes present in the signal.

**Figure 5:**
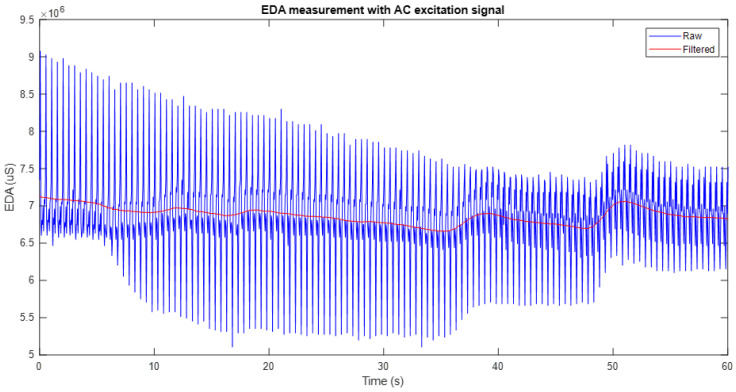
Application of a moving average filter to an EDA signal. The original signal (blue) is compared to the filtered signal (red).

Figure 5 shows an example of the application of the moving average filter to an EDA signal obtained with the system developed in this work under full-wave AC excitation. In the graph, the original signal (in blue) is compared with the filtered signal (in red), which shows a significant smoothing that reduces the noise without altering the main characteristics of the signal. This process preserves the general trend of the signal, facilitates the identification of relevant variations, and attenuates unwanted fluctuations, thus improving the quality of the measurement and its subsequent analysis.

It is important to note that the window filter used corresponds to one second of the data segment. Therefore, in cases where excitation frequencies higher than 20 Hz were used, such as 70 Hz and 100 Hz, it was necessary to increase the sampling frequency of the system up to 200 Hz to ensure adequate signal acquisition. Consequently, for these conditions, the applied filter window covered 200 samples, maintaining the time equivalence of one second.

#### 2.4.2. Phase Angle of EDA Signal

As mentioned in Section 2.1, the measurement of the phase angle (ϕ) allows for the analysis of the skin’s electrical properties by decomposing admittance into its two components: conductance and susceptance.

In this work, the method known as lock-in amplification [36] is implemented, which has been used in previous studies related to the analysis of EDA signals [5,17]. This technique is employed in applications where signals are weak and have a high noise level. Its principle of operation is based on generating a reference signal with the same frequency as the AC excitation signal used in the measurement, allowing for precise determination of the phase of the measured signal.

The process consists of multiplying the measured signal by two reference signals: one in phase and the other with a 90° positive offset. Typically, the reference signal is a sine wave, while the second is a cosine wave. Both reference signals must have the same frequency (*f*). Finally, a low-frequency pass filter is applied to isolate the components of the signal at the frequency of interest, removing any interference and ensuring that the resulting signal is free from noise. In this way, both the magnitude and the phase angle relative to the established reference can be obtained.

### 2.5. EDA Signal Components

The EDA signal consists of two components: tonic and phasic, whose decomposition allows for the analysis of sweat gland activity and its relationship with emotional, cognitive, and physiological states [4]. The tonic component reflects the baseline skin conductance level, including drifts and spontaneous fluctuations [5], and is key in monitoring autonomic neuropathy [12]. The phasic component captures rapid and transient responses to external stimuli [5].

Several analysis methods have been proposed for the separation of these two components, among which cvxEDA [37], based on convex optimisation techniques, and SparsEDA [38], which relies on sparsity criteria, stand out. Although both methods offer advantages, their performance may be affected by noise levels or signal quality. In this study, cvxEDA was selected due to its balance between noise robustness and ease of implementation, thanks to the libraries available in [39].

This work focuses on the analysis of the tonic component of the EDA signal, with the aim of evaluating the consistency of this measurement when obtained using DC and AC excitation signals. The purpose is to determine the correspondence between both stimulation methods and, thus, to assess the feasibility of using AC signals as a reliable alternative to traditional DC-based measurements. This comparison is relevant in contexts where it is necessary to minimise or avoid electrode polarisation effects in order to obtain more stable and accurate measurements. The possibility of using AC signals without compromising the quality of the SCL measurement can be very useful in applications related to user experience assessment in biomechanical systems [6,7] as well as in the adaptive control of rehabilitation therapies [8].

### 2.6. Experimental Procedure

An exosomatic approach was employed, in which skin conductance is determined by applying a voltage or current between two electrodes [5]. For the measurements using DC and positive half-wave AC excitation, a device based on the system described in [14] was used, while the measurements with full-wave AC signals were obtained using the system presented in this work.

The experimental procedure was divided into two stages. In the first stage, EDA signals obtained through DC and positive half-wave AC excitation at a frequency of 20 Hz were compared, with the aim of analysing the correspondence between both methods in terms of the tonic component of the signal. The second stage involved carrying out measurements using DC and full-wave AC excitation at frequencies of 5 Hz, 20 Hz, 70 Hz and 100 Hz. These frequencies were specifically chosen based on simulation results that explored the skin’s response across a broader range (from 1 Hz to 100 Hz) to evaluate field penetration and current distribution. By selecting representative frequencies within this range, the experimental setup enabled the comparison of SCL signal dynamics and the analysis of how the skin’s electrical behaviour evolves with increasing excitation frequency.

**Figure 6:**
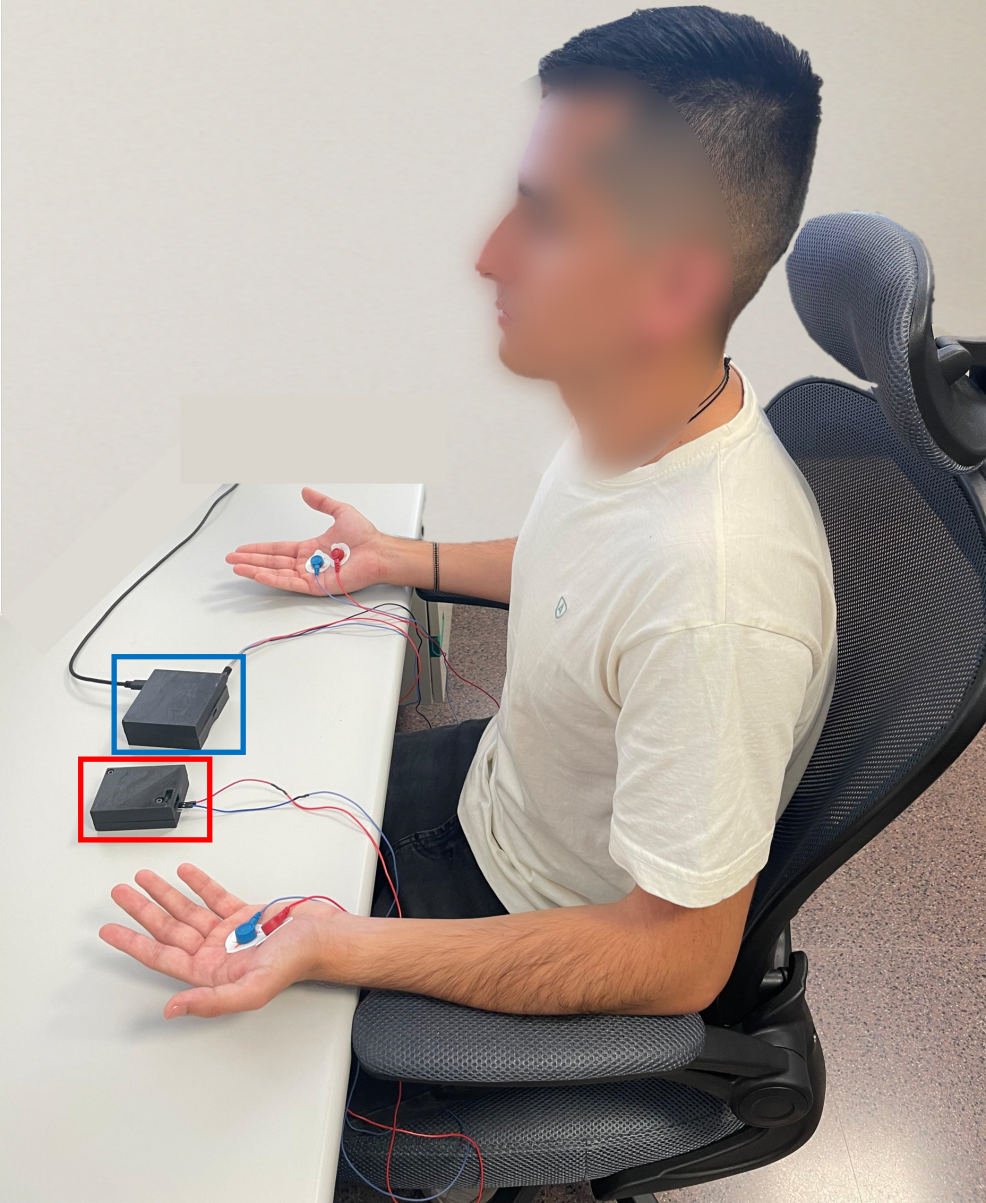
Experimental setup during EDA signal acquisition. The subject is simultaneously connected to the AC excitation device, indicated in blue, and the DC excitation system, marked in red.

Measurements were conducted on the palms of both hands, with DC excitation applied to the left hand and AC excitation to the right hand simultaneously. This setup ensured temporal synchronisation of the signals and allowed a direct comparison between the excitation methods. Figure 6 shows a participant during the data acquisition process. The image highlights the two devices used: the AC excitation measurement system is marked in blue, while the DC-based device is indicated in red.

The study included ten healthy adult participants (five female, five male) with an average age of 38 (±15) years. All participants were in good general health and reported no dermatological, neurological or cardiovascular conditions. Participants were excluded if they were taking medications that affect autonomic function, had visible skin damage in the electrode area, or had a diagnosed autonomic disorder. Participants were recruited via institutional invitation and provided written informed consent prior to taking part in the study. To ensure consistent signals and minimise environmental influences, all measurements were carried out in a quiet, controlled indoor environment with a room temperature maintained between 22 and 24 °C. Measurements were performed during the day (between 10:00 and 14:00) to minimise circadian variability.

During the experimental protocol, each participant was seated comfortably in a chair with their hands resting on a table to minimise involuntary movements. Before placing the electrodes, the skin was cleaned with isopropyl alcohol to remove any residues that could interfere with the measurement.

In the first experimental stage, the total duration of each acquisition was approximately 65 s per participant. In the second stage, each excitation frequency was applied for 180 s (3 min), with a minimum rest period of 2 min between measurements to reduce potential interference from external stimuli or spontaneous movements.

For signal acquisition, Dormo [40] Ag/AgCl electrodes coated with hydrogel were used, with an active area of 2 cm². After electrode placement, participants remained at rest for one minute to stabilise any emotional fluctuations or previous movements.

## 3. Results

The study was divided into two phases with the aim of comparing the characteristics of the SCL component of the EDA signal obtained by DC and AC excitation. In the first stage, a positive half-wave AC signal at a frequency of 20 Hz was used, while in the second stage, a full-wave AC signal was employed. During both phases, participants remained at rest and were not exposed to external stimuli in order to assess the baseline behaviour of the signal. However, the potential occurrence of spontaneous fluctuations associated with phasic responses (SCR), due to individual emotional factors, is acknowledged.

### 3.1. Experimental Stage 1

**Figure 7:**
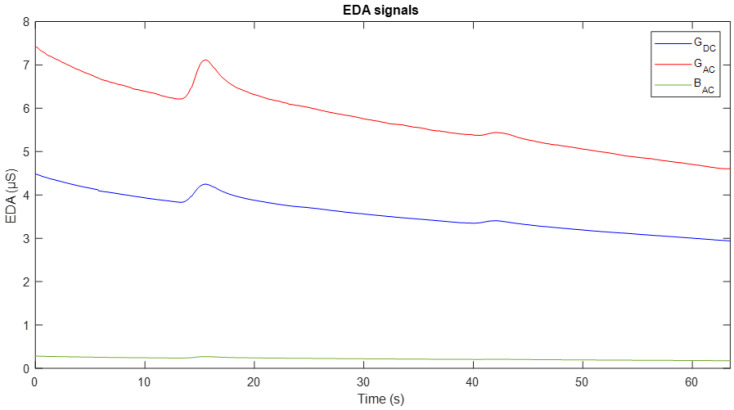
Experimental acquisition result corresponding to Stage 1. Conductance measured with DC excitation is shown in blue, and conductance with AC excitation in red. The susceptance component associated with the AC signal is represented in green.

Figure 7 shows an example of experimental acquisition during an interval of approximately 65 s. It shows three signals: the conductance obtained with DC excitation (G_*DC*_) in blue, the conductance with AC excitation (G_*AC*_) in red, and the susceptance component (B_*AC*_) in green.

A high correspondence is observed in the dynamics of the conductance measurements obtained by both methods. In general terms, the two conductance signals exhibit a decreasing trend over time, interrupted by slight fluctuations or increases around seconds 15 and 40. However, following these peaks, the signals resume their downward trend, which can be interpreted as an indication of the subject’s return to a baseline conductance state. Regarding the susceptance component, it follows a similar dynamic to G_*AC*_, although with lower magnitude, reflecting different aspects of the skin’s electrical activity.

**Figure 8:**
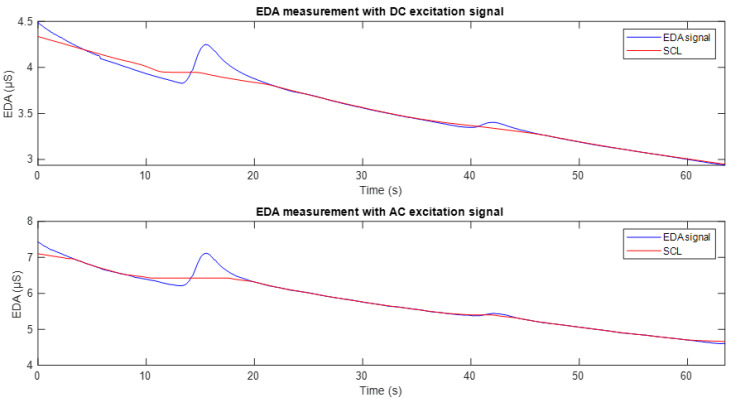
EDA signals obtained through DC and AC excitation (in blue), with their respective tonic components (SCL) estimated using the cvxEDA algorithm (in red).

Figure 8 shows the EDA signals obtained through DC and AC excitation, in blue, along with their respective tonic components (SCL), in red, extracted using the cvxEDA algorithm. This component reflects the basal level of sweat gland activity at rest. It can be seen that the fluctuations around seconds 15 and 40 are excluded in the estimation of the SCL component, allowing an analysis focused exclusively on the tonic variation. This representation is appropriate for the purpose of the study, which is to compare the SCL tracking between the two excitation methods.

**Figure 9:**
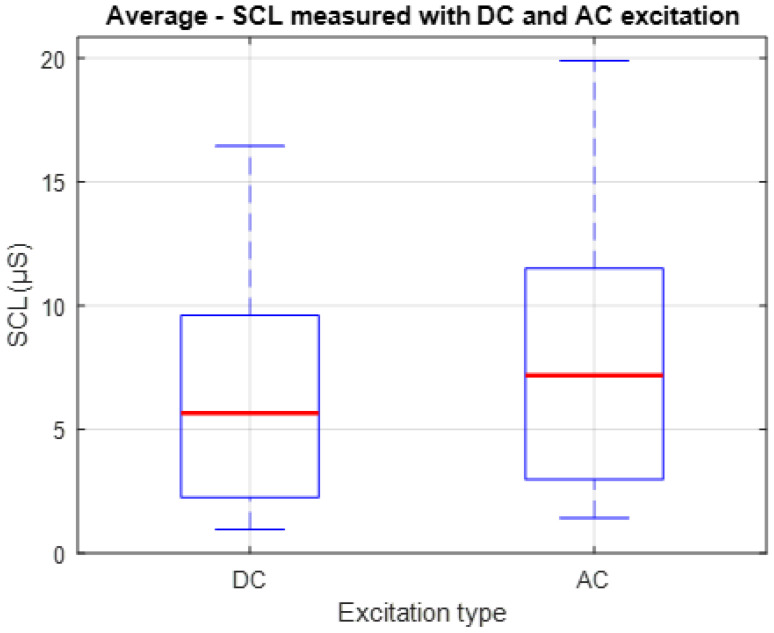
Box-and-whisker plot of individual mean SCL values obtained with DC and AC excitation during Experimental Stage 1. The red lines indicate the central tendency for each excitation method.

Figure 9 presents a box-and-whisker plot showing the individual mean values of SCL recorded from each of the 10 participants during Stage 1 of the experiment. Although the values obtained with AC excitation tend to be slightly higher in magnitude than those obtained with DC, the consistency in the shape of the distribution indicates that the baseline dynamics of the signal are preserved between both methods. This representation allows for the comparison not only of the central tendency, indicated by the red lines, but also the dispersion of the measurements, providing insight into the functional equivalence between both techniques for tracking the tonic component of the EDA signal under resting conditions.

To quantitatively compare the SCL signals obtained using the two excitation methods, the Pearson correlation coefficient was calculated for each of the ten participants, yielding an average value of r = 0.943. This indicates a high degree of similarity between the signals.

In terms of amplitude, the mean absolute SCL value was 6.359 ± 0.593 µS under DC excitation and 7.957 ± 0.645 µS under AC excitation. The root mean square error (RMSE) between the two datasets was 1.636 µS, reflecting the average difference in signal values between conditions.

### 3.2. Experimental Stage 2

**Figure 10:**
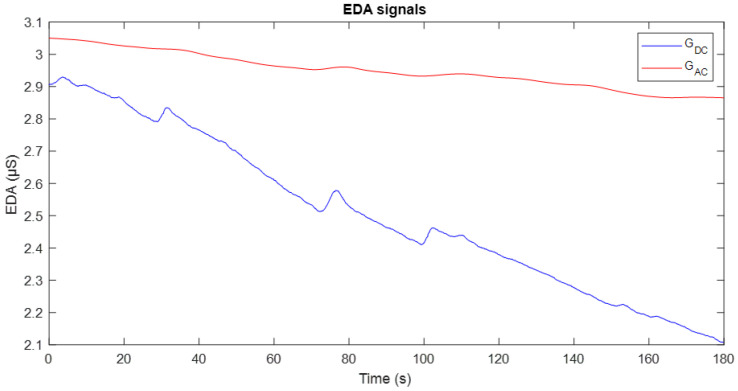
Temporal evolution of skin conductance obtained using DC excitation (blue) and full-wave AC excitation at 20 Hz (red).

The second experimental stage began with the acquisition of EDA signals using DC excitation and full-wave AC excitation at a frequency of 20 Hz. Figure 10 illustrates an example of the conductance evolution obtained through these two methods: the signal recorded with DC excitation is shown in blue, while the signal corresponding to AC excitation is displayed in red.

The G_*DC*_ signal exhibits greater variability over time and higher sensitivity to minor changes or stimuli, reflecting individual physiological responses. However, this apparent sensitivity is affected by electrode polarisation, which manifests as a progressive downward drift in the signal. In contrast, the G_*AC*_ signal demonstrates greater stability and reduced drift, which is particularly advantageous for long-term studies, as it minimises polarisation effects and enhances the quality of baseline recording.

**Figure 11:**
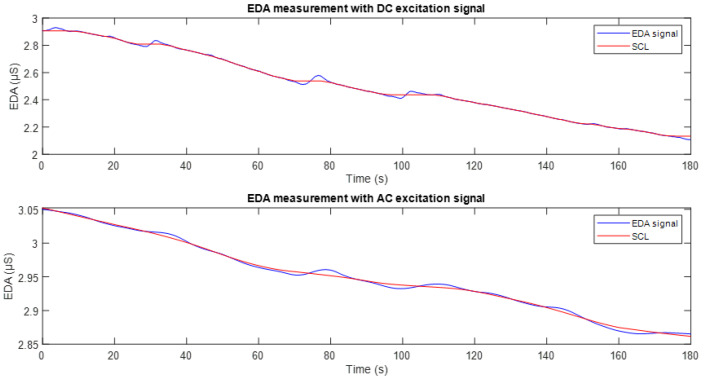
Conductance signals recorded with DC excitation (**top**) and AC excitation (**bottom**), shown in blue. In both plots, the tonic component (SCL) is included and represented in red.

Figure 11 shows the EDA signal recorded using DC excitation (top) and full-wave AC excitation (bottom). In both plots, the corresponding tonic component (SCL) is overlaid in red. It can be observed that in both cases, the SCL appropriately follows the baseline trend of the conductance signal. However, as can be seen in Figure 10, the signal obtained with DC excitation exhibits fluctuations and a downward drift, reflecting the influence of the polarisation phenomenon. In contrast, the SCL derived from the AC-excited signal shows higher stability and reduced drift, making it more robust and suitable for studies focusing on the tonic component of the EDA signal.

**Figure 12:**
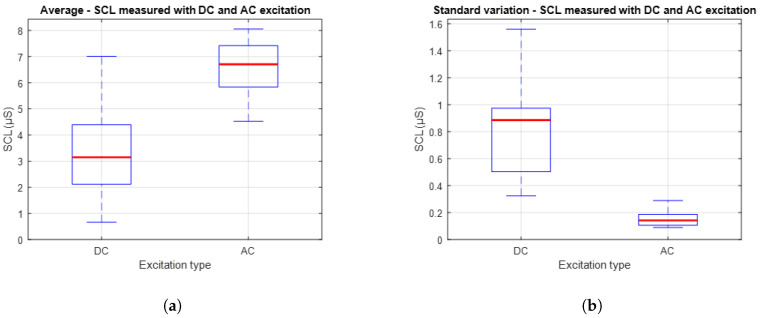
(**a**) Distribution of average SCL values for each type of excitation. The red lines indicate the central tendency for each excitation method. (**b**) Standard deviation of the average SCL values obtained with each excitation method. The red lines indicate the central tendency for each excitation method.

Figure 12 presents a comparison between the average and variability of the SCL signals obtained using DC and AC excitation. Specifically, Figure 12a shows the distribution of average values, while Figure 12b presents the standard deviation associated with these measurements. It is observed that AC excitation results in higher average SCL values compared to DC excitation. This outcome may be attributed to deeper penetration of the excitation signal into the skin layers. On the other hand, the plot on the right shows that the standard deviation of the signals obtained with AC excitation is lower than that recorded with DC. This suggests that measurements under AC excitation are more stable and consistent over time.

In the second experimental stage, which focused on signal stability over longer acquisition periods, the average Pearson correlation coefficient between signals obtained using DC and AC excitation was r = 0.758, indicating moderate similarity. The mean SCL was 3.345 µS for DC excitation and 6.575 µS for AC excitation. Meanwhile, the RMSE between the two signals increased to 3.612 µS, reflecting a more pronounced difference in amplitude. It is also notable that the average standard deviation of the AC-based signals was ±0.079 µS, indicating a high level of temporal stability.

**Figure 13:**
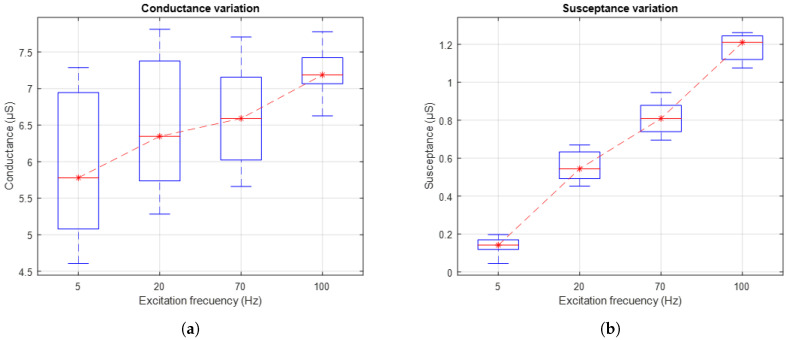
(**a**) Variation in skin conductance under AC excitation at different frequencies. The red lines indicate the central tendency of conductance variation. (**b**) Variation in skin susceptance under AC excitation at different frequencies. The red lines indicate the central tendency of susceptance variation.

Figure 13a shows the evolution of skin conductance, while Figure 13b presents the skin susceptance in response to AC excitation signals applied at different frequencies: 5 Hz, 20 Hz, 70 Hz, and 100 Hz. The box-and-whisker plots reflect the distribution of the values measured in the 10 subjects. The red dashed line connects the medians of each group, facilitating the visualisation of the general trend.

The results show an increase in both conductance and susceptance as the excitation signal frequency increases. This trend suggests that higher frequencies promote greater signal penetration into the different layers of the skin. Consequently, the use of higher frequencies allows for a more comprehensive characterisation of the skin’s electrical properties, highlighting changes in its electrical components that depend on penetration depth.

## 4. Discussion and Conclusions

Previous studies have reported the use of skin conductance level (SCL) signals, derived from EDA, in the context of user adaptation and experience evaluation in biomechanical applications [6,8,40]. Traditionally, these measurements are performed exosomatic method using DC excitation [15]. However, this approach presents a significant limitation related to polarisation at the electrode–skin interface [5], which causes signal drift and variability in measurements, making it difficult to accurately represent real changes in conductance, especially in long-term recordings.

For this reason, it is relevant to explore EDA measurement using AC excitation, as this technique, by cyclically inverting the polarity, reduces the polarisation effect [5]. This improvement can result in more stable and representative signals, which is especially valuable for the development of wearables systems applicable in biomechanical environments, such as the device proposed in this work.

In the case of AC excitation, it is essential to understand the skin’s bioelectric response in order to select an appropriate excitation frequency. In this context, it has been reported that low frequencies are particularly useful for assessing conductance sensitivity associated with sweat gland activity [35]. More specifically, it has been indicated in [17] that frequencies between 10 and 100 Hz are the most suitable for this type of analysis.

Thus, the simulation of the skin’s electrical response presented in Section 2.2 provides key information on the current distribution across its different layers. The results obtained support the findings of previous studies, demonstrating, as shown in Figure 2, that low-frequency AC signals manage to penetrate into the dermis, the layer that houses the highest density of sweat glands [28]. This penetration not only helps mitigate the polarisation effect but also allows access to information related to the skin’s capacitive component, that is, the susceptance. Additionally, the simulation results show how the interaction of the electric field between the electrodes in contact with the skin increases as the excitation frequency rises.

The experimental phase of this study highlights the importance of keeping the subjects in a relaxed state during the measurements, in order to analyse the basal response of sweat gland activity from the SCL signal. To achieve this, the tests were conducted under controlled conditions, minimizing the influence of external factors on the participants’ physiological response.

Under this approach, the initial proposal was to compare the EDA signal obtained using DC excitation with that acquired using half-wave AC excitation, i.e., with only the positive component at a frequency of 20 Hz. As observed in the results (Figure 7), the conductance signal obtained with DC and AC excitation shows a decreasing trend over time, accompanied by small fluctuations. This same dynamic is reflected in the susceptance component associated with the AC signal, which aligns with the findings reported in [17,35], where a high correspondence between the measurements obtained using both excitation methods is evident. It is important to note that, since it is a half-wave AC signal, with no negative component, there is no polarity inversion. Consequently, the polarisation effect at the electrode–skin interface is not mitigated, resulting in a downward drift similar to what is observed with DC excitation. The similarity observed between the SCL signals obtained using the two excitation methods was further supported by correlation analysis, which revealed a strong temporal relationship between participants. This suggests that despite the recognised limitations of half-wave AC in mitigating drift, both techniques can accurately detect consistent tonic EDA activity, indicating the presence of similar underlying physiological processes.

On the other hand, regarding the distribution of the SCL records obtained through each excitation method, shown in Figure 9, a high consistency is observed between both, despite the differences in magnitude. This difference can be attributed to the use of wet electrodes, which, in addition to facilitating the penetration of the electrical signal into the deeper layers of the skin, also allow greater access to the sweat ducts in the superficial layers [41].

The results indicate a low overall discrepancy between the signals obtained using the two excitation methods, which reinforces the correspondence between the measurements. Although differences in absolute amplitude were observed, the temporal dynamics of the SCL signal remained consistent across methods. This aligns with the primary objective of this experimental stage, which is to evaluate the equivalence in tonic signal behaviour. Nevertheless, these results allow us to conclude that the measurement system used, based on the proposal of [14], offers a high level of agreement between the two excitation methods.

In the second experimental phase, a full-wave AC excitation signal was used. The results presented in Figure 10 clearly show the effect of this signal on the conductance measurement. Compared to the signal obtained in the first stage, a greater temporal stability is evident, with reduced drift and less sensitivity to minor stimuli, which is consistent with the findings reported in [5]. This lower variability suggests that the SCL signal obtained through full-wave AC excitation is less modulated over time, reflecting a more stable behaviour. Although the correlation between the signals from the two excitation methods was lower than in the first phase, this was mainly due to the suppression of drift in the AC-based signals. When this artefact is present in both signals, it increases the correlation. This supports the idea that, as well as improving stability, full-wave AC excitation provides a clearer representation of tonic EDA activity.

Regarding the magnitude of the measurements represented in Figure 12a, consistent differences are observed when compared to those recorded in the first experimental phase. These differences can be attributed to both the use of wet electrodes [41] and the greater ability of the AC excitation method to penetrate deeper layers of the skin [42], which allows for a more representative capture of the galvanic activity. Additionally, AC excitation tends to generate cleaner signals by minimizing the capture of noise and interference, contributing to greater stability in the measurements. These effects are reflected in the amplitude gap between the DC and AC signals. This further reinforces the superior performance of the AC-based acquisition approach when it comes to capturing tonic EDA.

Finally, regarding the temporal stability of the signals, even under controlled conditions without external stimuli, the standard deviation analysis, presented in Figure 12b. The reduced variability observed in the AC-based measurements suggests that this method is less susceptible to the minor fluctuations and baseline drift that often affect DC recordings. This supports the idea that using full-wave AC excitation provides a more stable and reliable way of capturing the tonic EDA component. This makes it a more robust approach for long-term monitoring or wearable applications, where consistent signals are important.

This study focused on analysing basal EDA under resting conditions. Therefore, no external stimuli were applied to elicit phasic responses. While this design enabled the isolation and evaluation of signal stability and quality, it limited the assessment of dynamic responses, such as the SCR. It would therefore be appropriate to propose an experimental scenario focused on evaluating the phasic component of the EDA signal, as suggested in [20]. This would enable the system’s sensitivity to be assessed not only to tonic variations, but also to discrete events or specific stimuli. This is essential for applications in ambulatory contexts or during active interaction with biomechanical devices, as signal robustness and performance under less controlled conditions become critical in these scenarios. Future work should also incorporate a broader pool of participants and structured stimulus protocols to evaluate the system’s responsiveness to the SCR in more diverse physiological situations.

The results obtained from the variation in excitation frequency reveal an increasing trend in both skin conductance and susceptance, as shown in Figure 13. This trend aligns with findings reported in [19], which indicate that increasing the frequency of the excitation signal leads to a rise in the system’s phase angle, thereby reflecting a greater involvement of the capacitive component in the skin’s bioelectrical response. Although frequencies above 20 Hz have proven effective in capturing relevant information related to sweat gland activity, the simulation results presented in this study suggest that lower frequencies, close to 1 Hz, may also constitute a viable strategy to mitigate the polarisation effect at the electrode–skin interface. This approach is particularly suitable when the primary objective is to minimise noise and enhance the stability of baseline recordings, without necessarily requiring high-resolution characterisation of the phasic response. In this regard, future studies could more systematically explore the trade-off between excitation frequency, signal quality, and the specificity with which the physiological activity of interest is represented, particularly in applications where recording stability is prioritised over sensitivity to rapid or transient events.

Overall, the results of this study confirm the feasibility and relevance of using AC excitation signals for the measurement of EDA, particularly for the estimation of the tonic component (SCL). The systematic comparison between DC excitation, half-wave AC, and full-wave AC demonstrated the advantages of the latter in terms of increased signal stability, reduced drift, and mitigation of electrode polarisation effects. Additionally, the analysis of susceptance provided complementary information for characterizing the skin’s bioelectrical response, particularly with regard to sweat gland activity located in deeper layers.

Moreover, the findings support the use of wearable systems, such as the one developed in this work, for real-time applications, representing a significant improvement in EDA measurement in ambulatory, clinical, and human–device interaction contexts within biomechanical applications.

This study demonstrates the feasibility of using full-wave AC excitation for EDA measurement. However, there are several areas that require further research. A key next step is to evaluate the system’s response to phasic EDA components under stimulus-driven conditions. Additionally, integrating wireless communication (e.g., Bluetooth) would enhance the system’s portability for real-world use. Testing should be expanded to include a more diverse group of participants, including clinical populations, to enhance generalisability.

Finally, while the focus here is on skin conductance, using AC excitation may capture responses from deeper tissues, similar to those obtained using bioimpedance methods. Future research should explore this possibility, particularly in anatomically complex body regions, and compare the results with those obtained using standard bioimpedance techniques.

## Data Availability

Data available on request due to restrictions.
